# Practicing Surgeons’ Perception of Barriers to Palliative Care Delivery in British Columbia

**DOI:** 10.7759/cureus.58061

**Published:** 2024-04-11

**Authors:** Kadhim M Taqi, Christina W Lee, Jenny W Zhang, Philippa Hawley, Rona Cheifetz

**Affiliations:** 1 Department of Surgery, University of British Columbia, Vancouver, CAN; 2 Department of Medicine, University of British Columbia, Vancouver, CAN

**Keywords:** palliation in surgery, access to healthcare, access to palliative care, general surgery, palliative care

## Abstract

Background: Utilization of palliative care remains low among surgical patients. We aim to characterize general surgeons’ perceptions of barriers to access palliative care in British Columbia (BC).

Methods: Semi-structured interviews were carried out with a total of 11 surgeons in BC. Interviews were transcribed for thematic analysis via interpretive description. Dominant themes were identified and agreed upon between the authors. Results: Several barriers were identified, which include system and institution, communication and surgical workflow barriers. At the system and institutional level, there were difficulties accessing patient information and continuity of care. Themes in the communication included patient misconceptions about palliative care and communication challenges with consulting services. Surgical workflow barriers influenced the overall perceived role of surgeons when caring for patients with palliative care needs.

Conclusion: Understanding surgeons’ perspectives on barriers to palliative care is an important step in changing management. This can aid in the development of strategies that ease access to palliative care.

## Introduction

Compared to patients primarily under the care of medical specialists, those under surgical services are less likely to receive specialist palliative care (SPC) during the final year of their lives [1,2]. This incongruity persists even though 10-20% of all surgical procedures are done with palliative intent, i.e. to improve symptoms or manage complications without the potential to cure [3-5]. A variety of factors have been described to account for these differences [1,2,6,7], with the most common limitation being the absence of SPC services available [1]. Unfamiliarity with palliative care skills and the limited focus on palliative care competencies in surgical training programs are strong contributing factors [6]. Other barriers to delivering palliative care in surgical patients include difficulties in predicting outcomes, patient and family expectations, inadequate discussion and documentation of goals of care and patient preferences [2,7]. It is important to understand that access to palliative care does not always require a specialist, and many patients’ palliative care needs can be met by primary care, in both rural and urban settings [8].

Inadequate assessment and recognition of palliative care needs in surgical patients can lead to substantial physical, emotional and psychological consequences for patients and their families [1,6,9]. It can also lead to goal-discordant interventions being done, causing patients to remain in acute care longer than necessary [10]. Previous studies on these issues have focused primarily on medical specialist experiences, while the surgeons’ perspectives in select health systems and cultures have been explored to a lesser extent [11]. There are no prior reports describing Canadian general surgeons’ experiences with palliative care delivery in the primary or specialist context in managing patients with palliative needs.

The Canadian health system is a universal, publicly funded healthcare system [12]. This allows for more equitable access to physicians and healthcare facilities [13]. Services however vary depending on resources available from one site to another, with SPC services not generally as available in rural settings as in urban settings. The aim of this report is a narrative of practicing general surgeons’ perspectives of barriers unique to the experiences in access to palliative care for patients under the care of general surgeons in BC.

## Materials and methods

This study is an exploratory, qualitative study investigating the perceptions of general surgeons’ experiences with palliative care services in BC. It is part of a larger ongoing study that investigates the general surgical experience with palliative care in different aspects, such as education, training and referral systems. This is the first published data from this ongoing project. The study was approved by the BC Cancer Research Ethics Board (REB Ethics ID: H20-01573).

All practicing general surgeons in BC were recruited via email through membership with the British Columbia Surgical Society (BCSS). All participants were general surgeons practicing in the province of BC (n = 11). Participation was not limited to active surgeons only, as retried surgeons were also contacted and included in the interviewed population. The study population was not limited to academic centers as it included surgeons from rural and community hospitals as well to establish a broader perspective from all over the province. Visiting surgeons and surgeons non-affiliated with the BCSS were excluded. Voluntary participation was elicited via a computer-based survey followed by individual semi-structured interviews conducted by the authors (CWL, KT and JZ). The semi-structured interview questions were developed by the authors and were all open-ended questions and started with a general question about the perception of palliative care in the surgeons' experience. The domains and categories included knowledge and prior experience, education and training, communication and goals of care and barrier and challenges (see Appendix). Due to the COVID-19 pandemic and logistical considerations of distance, all interviews were completed virtually and audio-recorded. The recording was then transcribed verbatim for thematic analyses using an online transcription application (otter.ai) using a secure login account. All transcripts were then uploaded to a password-protected Cloud account accessible only to the investigators. Alphanumeric identifiers were used to protect participant confidentiality. Transcripts were not returned to participants for review. All interviews were completed from November 2020 to March 2021.

Content and thematic analyses were performed using the interpretive-descriptive paradigm [14]. Data were analyzed among the authors through an iterative process of coding to identify dominant, clinically, socially and psychosocially recurrent perspectives and concepts without the use of qualitative software. The theme identification was ongoing throughout the process of analysis. The codes and emergent themes were established through multiple rounds of conversations and comparisons and were selected to encompass the diverse range of perspectives gathered from the participants, highlighting the depth of insights they provided. The coding process and final interpretations were re-visited with differences resolved through discussion and consensus by using insights from the field.

## Results

Eleven practising general surgeons in BC participated in semi-structured interviews. Table 1 shows the demographics and surgical experience of the participants. Three main themes emerged with respect to surgeons’ perceptions of barriers to palliative care service (PCS) access with numerous subthemes within each thematic category:

**Table 1:** Demographics, practice and surgical experience of the interviewed surgeons

Category	Details n (%)
Gender	Male: 4 (36.4)
	Female: 7 (63.6)
Hospital setting	Rural: 6 (54.5)
	Urban: 5 (45.6)
Years of experience	≤10 years: 3 (27.3)
	11-20 years: 3 (27.3)
	>20 years: 5 (45.6)
Specialty	General surgery: 10 (90.1)
	General surgery subspecialty: 1 (9.9)
Practice	Retired: 2 (18.2)
	Practising: 9 (81.8)

System and institutional barriers

System and institutional barriers are related to the healthcare system across the province and the challenges arising with patients receiving care across different regions of the province, with variable surgeon knowledge of currently available resources. The subthemes identified included access to patient information, continuity of care after discharge and availability of resources.

Surgeons described practical difficulties in accessing patient information across different healthcare authorities. In BC, every health authority governs, plans and delivers healthcare services within their geographic areas, but BC does not employ a province-wide universal electronic medical record (EMR) system. While institutionally generated results are shared via CareConnect (a combined secure network), this does not include notes from private facilities including anything generated from primary care or non-hospital-based specialist offices. This issue becomes challenging, especially in the setting of an emergency presentation. Surgeons felt that when these patients present to the emergency department and with time constraints, it is hard to extract the patient health journey and identify the goals of care and whether these were discussed before, and if so, where to find the documentation, as well as challenges with data access across different EMRs.

Surgeons also described the limited availability of resources related to palliative care, such as residential care, home care nursing, occupational therapy and counselling. This can be challenging in communities where priorities are directed at triage for acute problems. This was reported more by surgeons based outside the major urban centres. In the surgeons' opinion, this is complex as it is related not only to the availability of resources and funding but also to the difficulties of having specialized care in rural settings where the care is more generalized.

Table 2 summarizes the subthemes identified in the system and institutional barriers level with some example quotes from the surgeons.

**Table 2:** Subthemes identified in the system and institutional barriers level with some example quotes from the surgeons.

Theme/subtheme	Quotes
System and institutional barriers	
a) Access to patient information	'It takes me lots of time sometimes to gather as much information as I can about what they've been told in the past. So that I'm not dropping a bomb on them telling them they have cancer!' - Surgeon 10
	'Patients come to the emergency, and it is hard at that time to deep dive into the whole treatment program and know the overall goals of care.' - Surgeon 2
b) Continuity of care after discharge	'Availability and variability of services in different regions after discharging the patients is challenging. However, sometimes people who live in more rural areas, the nursing staff are more experienced, and hence that responsibility falls on them.' - Surgeon 11
	'I often get consulted about patients who had a major event and there was no document about goals of care.' - Surgeon 8
c) Availability of resources	'I practice in a rural area, so we don't have tons of services! We don’t have palliative care but we do have a hospice; however, everything is kind of limited and a little bit cobbled together.' - Surgeon 9
	'… availability and variability of services in different regions after discharging the patients itself a challenge.' - Surgeon 4
	'… funding is not readily available for supporting the palliative service.' - Surgeon 7

Communication barriers

These barriers are related to professional communications within healthcare teams and interactions with patients and their families. The subthemes identified included the patient's and families’ perceptions of palliative care and challenges in communication with the patient's primary healthcare teams. Surgeons described difficulties communicating with their healthcare colleagues, patients, and their families about the patients' palliative care needs after discharge. This was mostly because of the lack of a core caring team that would be the centre of patient care, especially if they did not have a family doctor. Communicating discharge planning and care plans becomes a challenge and patients and events can fall in between the cracks.

Furthermore, patients and families may not have a clear understanding of the natural history and prognosis of the disease. In an emergency setting, this can be difficult when initiating discussions regarding the palliative approach to care. Establishing goals of care during the early stages of the disease can help patients and their families prepare for future discussions regarding palliative care needs especially when experiencing acute deterioration. Surgeons found that starting the whole discussion and trying to explain the journey of care for the patients and their families is challenging, especially in the acute setting. This gap is further widened in the surgeon’s opinion when their communication has not been clear from the beginning in regard to the prognosis and makes establishing the goals of care in the acute setting a difficult conversation.

Table 3 summarizes the suthemes identified in the communication barriers level with some example quotes from the surgeons.

**Table 3:** Subthemes identified in the communication barriers level with some example quotes from the surgeons.

Theme/subtheme	Quotes
Communication barriers	
a) Who can I discuss the case with?	'It would be helpful if I was able to contact someone about the palliative case, and maybe discuss and review to reach an informed decision.' - Surgeon 1
	'... and if they're coming from different hospitals, or they're being seen by different physicians, everyone's going to have their own idea of what can be done, but there should be a core team that decides about the plan.' - Surgeon 4
	'... there's often inappropriate delay just because there's not somebody dedicated to a palliative care service.' - Surgeon 10
b) Patient and family misconception about palliative care	'There's lots of misconceptions about what palliative care means, especially when you bring it up with a cancer patient who is at present as well and is not imminently dying. There are also lots of family dynamics that can come up as well.' - Surgeon 6

Surgical workflow barriers

These barriers were identified by practising general surgeons related to the constraints of day-to-day scheduling and time management affecting their patient care. The subthemes identified included surgeons’ late involvement in the disease process, workflow challenges and uncertainty about the surgeon’s role in the delivery of PCSs to their patients.

A distinguishing characteristic of palliative care for surgical patients is that surgical consultation is often delayed until the patient experiences a new complication or progression of their disease that has not been previously addressed. Surgeons believe that there are some issues and presentations that are predictable (i.e. malignant bowel obstruction in patients with abdominal malignancies) where the probabilities should be discussed with patients early in the course of the disease in order to align their goals and allow them to understand their disease and prognosis.

Moreover, surgeons have busy schedules filled with surgical, endoscopic and clinical responsibilities in addition to other roles, such as academic and administrative responsibilities. Hence, stringent time management and special allocation of time for goals of care discussions with patients are limited.

The role of surgeons in caring for patients requiring PCSs can be highly variable and often ill-defined. The specific responsibilities of a surgeon may depend on the resources and support available within a healthcare authority. In some authorities, a designated liaison or dedicated palliative healthcare provider/team may be available to help coordinate the care of patients. This ensures that the patient's physical and psychological needs are being met and that their care is tailored to their individual goals and preferences. In other healthcare settings, the responsibility for providing palliative care support may fall entirely on the surgeon, which can be challenging.

Table 4 summarizes the subthemes identified in the surgical workflow barriers level with some example quotes from the surgeons.

**Table 4:** Subthemes identified in the surgical workflow barriers level with some example quotes from the surgeons.

Theme/subtheme	Quotes
Surgical workflow barriers	
a) Busy morning workflow	'It often comes up at a time in the interview, where maybe all of a sudden, you are pressed for time. Though it's not appropriate to rush through these discussions, I know I've rushed through it a little bit!' - Surgeon 7
	'A lot of the stuff happens in the middle of the morning and that’s a challenge for us as a surgeon. Everybody's challenged for time.' - Surgeon 2
b) What is the role of the surgeon in patients with palliative care needs?	'In smaller communities where palliative or hospice service isn’t available, nursing support becomes a very important concept for these patients.' - Surgeon 5
	'I don't really see my role trying to mobilize all the support resources for palliative patients as I just don't have the time, so we consult the nurse.' - Surgeon 3
c) Are surgeons involved too late?	'Surgical care to palliative patients basically is something that comes in like at the 11th hour when they're pretty much on death's door and then the surgical team gets involved.' - Surgeon 3
	'One of the issues I encounter not that infrequently is that we get consulted at the last moment. And sometimes it doesn't sound like there's been a whole lot of discussion around the goals of care or treatment plan until the very end.' - Surgeon 9

## Discussion

This is the first study exploring the perceptions of palliative care delivery for surgical patients in BC as a uniquely Canadian experience. Multiple perceived barriers to accessing PCSs for their patients were described by general surgeons in BC, including system and institutional barriers, communication barriers and surgical workflow. Additional barriers identified in other studies among healthcare providers include a lack of awareness about the benefits of palliative care, a general focus on curative treatments rather than symptom management and a reluctance among surgeons to initiate discussions about end-of-life care [15]. Studies have also observed that surgeons’ attitudes and the 'cure culture' that dominates in surgery lead to the tendency to initiate palliative care referrals only after all curative options are exhausted [16]. Blumenthal et al. in 2021 surveyed 46 surgeons from various health settings in Michigan evaluating barriers for palliative care use among surgical patients. Some of the barriers identified included surgeons’ knowledge and attitude, which included the surgeon’s 'fixer' identity [9]. Surgeons reported that this often influenced relationships with patients and families as conversations around palliative care can be perceived as the surgeon giving up. However, surgeons felt that open communication with patients and their families often helped in building trust and rapport during difficult conversations especially when palliative care was considered as a core component of communication rather than an adjunct [9]. Suwanabol et al. surveyed 131 surgeons and showed that communication between teams was described as a 'large or huge' barrier to PCS access by more than 50% of the surgeons. This was felt to be largely due to unrealistic expectations of the referring service about patient prognosis or the effectiveness of treatment when communicating with the surgical team [2]. Our study also supports the identified issue of inadequate communication between teams and healthcare providers regarding patients’ goals of care.

One part of the communication barrier significantly described by BC general surgeons was the difficulty encountered in managing patients’ and families’ misconceptions of the goals of palliative approaches involving surgical interventions. When the disease advanced suddenly, patients and their families struggled with emotional adjustment, often with hopeful expectations of recovery. This, in turn, made establishing goals of care more difficult [17,18]. Surgeons in our study attributed patient misconceptions of PCSs to poorly delineated roles and responsibilities within the care team. Even when palliative care referral was agreed upon with the patient and family, factors such as initiation and coordination of care, patient and family meetings and follow-up plans were areas of confusion and conflict depending on the health authority and the resources available. This is a well-reported phenomenon, where confusion about role responsibilities adds to patient and family confusion and may lead to miscommunication regarding plans of care when multiple providers are involved [6]. Another challenge identified by the surgeons in our study was the lengthy decision-making process regarding palliative care versus curative interventions, which was time-consuming and often required multiple family meetings. The ability to reach a decision was often complicated by patients' and families’ misconceptions about the goals of palliative care and surgical interventions, as discussed in the study's findings [2,9].

Another barrier that was reported by some of the surgeons in our study was the lack of formal training and education during surgical residency. Surgeons often reported limited knowledge of both palliative care approaches and specialty services, which they attributed mainly to limited education and training surrounding palliative care [9]. The absence of knowledge, comfort and familiarity regarding opportunities for palliative care delivery with minimal or no training was a major barrier to access to PCSs despite the service availability [2]. This aspect sheds light on the importance of integrating palliative care in surgical training in the formal sense of established curricula in addition to less formal formats typically seen through role modelling [9]. Amini et al. surveyed fellows in their surgical oncology training and showed that more than a third reported no exposure to a palliative care specialty service during their fellowship. The surgical fellows felt the quality of their palliative care education was poor compared to other aspects of their training, and around 66% of them described insufficient training in palliative care communication and end-of-life symptom management [3]. Another study focusing on surgical residents interviewed 18 surgical residents to evaluate delays in palliative care referrals among surgical patients. Surgical residents expressed challenges in predicting patient outcomes and verbalizing this to both surgeons and families augmenting this uncertainty in seeking PCSs [6]. While some informal educational methods remaining in place include identifying mentors and undergoing additional training courses [2], efforts in surgical programs are ongoing to establish a palliative care curriculum with many programs having palliative care as part of their training objectives [9,19]. In 2000, the Accreditation Council for Graduate Medical Education (ACGME) and the Surgical Council on Resident Education (SCORE) included palliative care material and educational requirements in surgical residency training. The American College of Surgeons also produced a workbook intended as a guide for surgical residents in 2008 [20]. The Royal College of Physicians and Surgeons of Canada (RCPSC) also list palliative care as one of the main competencies now in their general surgery specialty training objectives and integrate palliative care as part of the CanMEDS framework [21]. The general surgery residency program at the University of British Columbia (UBC) lists palliative care within the goals and objectives of specific rotations, including surgical oncology and thoracic surgery [22].

Surgeons in our study also felt that their contribution and opinion were requested too late in the patient’s disease, and by then, the opportunity to provide insight or utilize other allied services for symptom management felt untimely. This was more challenging in the emergency setting given the time constraints and the high-pressure and high-stake environment. Hence, these observations in BC support the well-established benefits of the utilization of a multi-disciplinary team in conducting culturally sensitive and compassionate family meetings early following admission to improve patient care, ease familial burdens and align expectations with patient goals [17]. Earlier goal-directed care discussions in patients with predicted disease progress (i.e. malignant bowel obstruction in peritoneal carcinomatosis) could aid management [5]. Timely communication and discussions about goals of care with utilizing PCS even in the early inpatient settings tend to reduce suffering in the last days of life and provide patients with more accurate prognostic information [1].

Some studies have evaluated the role of prognostic tools and nomograms to facilitate palliative care discussions with patients and families [11]. In the implementation of a 'trigger' system, specific criteria such as duration of stay, age and life-threatening comorbidities trigger a proactive referral to PCSs. This method has been utilized in ICU patients and is currently being evaluated in acute surgical patients. It has been shown in ICU patients to reduce the utilization of resources without changing mortality while increasing the involvement of palliative care specialists for critically ill patients and families in need [17]. Using triggers associated with mortality and hospital readmission may be a possible way to identify earlier patients who may benefit from hospice or palliative care [1]. Better integration of palliative care routinely into the care of acute surgical patients not only in the emergency setting but also post discharge could also facilitate future advanced care planning [5,17]. This setup could be beneficial to practising surgeons with limited resources and address challenges that were raised, such as limited access, continuity of care and surgical workflow barriers.

There are several limitations to our study. First, although we aimed to capture as a wide range of surgeons' opinions as possible across the province, some unique experiences may not be represented. Second, this study only involved practising general surgeons and did not perceive the perception of other surgical team members, including residents or health allied services. However, in many of the community hospitals, the surgeons work independently, and we wanted to view the spectrum of barriers across the whole province. While the study sample is small, it did represent a spectrum of surgeons across different practice settings with different years of practice experience. Finally, due to the study being part of a larger qualitative review, many of the interview questions were open-ended, allowing for larger-than-anticipated information and responses, which the investigators will utilize for further work in the future.

## Conclusions

This study identified barriers to access PCSs as perceived by practicing general surgeons in the province of BC. The insights gained from this qualitative study can be used to develop strategies to create collaborative relationships with interdisciplinary palliative care teams with the shared goal of reducing harm from surgical interventions that are inconsistent with patients’ goals of care and facilitating the transfer of care out of the acute care environment.

## Appendices

Semi-structured interview guide

Study: Practicing Surgeons’ Perception of Barriers to Palliative Care Celivery in British Columbia Time allotment: 30-60 minutes Modality: Zoom/Telephone (Zoom call-in for audio-recording tool)

**Interviewers:** Christina Lee, MD Kadhim Taqi, MD Jenny Zhang

INTRODUCTION

Thank you for agreeing to participate in our interview today. My name is _________ and I am a member of the research team. I’d like to talk to you to learn about your experiences with palliative care in BC. I’m specifically interested to learn about the ways in which you communicate with your patients during challenging situations, as well as the services and resources available to you in caring for seriously-ill patients.

This interview should take approximately 30 to 60 minutes to complete, but can range from 20 minutes to one hour, depending on our conversation. Please do not hesitate to let me know if you are needing to end the interview at any point, and we can arrange for another more convenient time for you.

Also, everything you tell me will remain confidential. If it is ok with you, I will capture our interview on a voice recorder for the purpose of transcription in order to review and analyse findings from our study. The results in the end will be generalized and your anonymity will be kept confidential throughout this entire process.

An informed consent was emailed to you ahead of time for your review. Thank you for completing this.

Do you have any questions before we start?

**Table 5:** Interview questions developed by the authors

Question	Prompt	Category
Tell me what palliative care means to you?	- What services come to mind with palliative care? - What kind of responses do you get from patients, their caregivers and other surgeons when palliative care comes to mind?	Knowledge & Experience
Can you describe situations in which you would consider either a palliative care consult or triggers that would suggest a patient would benefit from any of the palliative care services? What are your thoughts on the role of palliative care for non-imminently dying patients?	- Things that worked well and things that did not work well - What does the palliative care team look like?	Experience & Education Training
Tell me about an experience you had with a patient who underwent an elective or emergent operation and suffered uncontrollable postoperative symptoms?	- How did you decide how to break bad news? - How did you feel after this conversation?	Experience & Management
Ask if interviewee doesn’t mention an experience with death/EOL Tell me about an experience with a patient who died or was near the end of life? Here, we’re less interested in the clinical details, and more in your approach and the processes involved. From those experiences, can you tell me what went well, and what didn’t?	- How and when do you decide to change the focus of care on quality of life rather than life-prolongation? - How did this experience make you feel?	Experience & Management
How do you talk to your patients before surgery for not being able to fix the problem or the potential for an unexpected event from surgery?	- What sorts of things do you consider in planning this type of conversation? What challenges do you face? - Do you use any conversation guides or surgical assessment/communication tools?	Communication & goals of care conversations
What do you view as the biggest challenge or barrier to providing palliative care as the surgeon?	- Comfort level – Can you tell me about your comfort level in discussing prognosis and end of life care or goals of care? - Demands of busy clinical practice - Are there aspects of your practice that make this challenging? - Any Institutional issues, resource availability, accessibility ?	Perceptions
